# SBMLPkgSpec: a LaTeX style file for SBML package specification documents

**DOI:** 10.1186/s13104-017-2788-1

**Published:** 2017-09-06

**Authors:** Michael Hucka

**Affiliations:** Department of Computing and Mathematical Sciences, California Institute of Technology, 1200 E. California Blvd., Pasadena, California 91125 USA

**Keywords:** SBML, XML, Data formats, Software, Simulation, Systems biology, Computational biology

## Abstract

**Objective:**

The Systems Biology Markup Language (SBML) is a popular open format for storing and exchanging computational models in biology. The definition of SBML is captured in formal specification documents. SBMLPkgSpec is a LaTeX document style intended to fill the need for a standard format for writing such specification documents.

**Results:**

Specification documents for SBML Level 3 extensions (known as *packages* in SBML) are made more uniform with the use of a standard template. SBMLPkgSpec is a LaTeX class that provides a document framework for SBML Level 3 package specifications, to simplify the work of document authors while improving the overall quality of the family of SBML specifications.

## Introduction

The Systems Biology Markup Language (SBML) is an XML-based (Extensible Markup Language) format that has become a community standard for the storage, communication and interchange of models in systems biology [1, 2]. The format has evolved in a community-driven fashion, with contributions from dozens of people worldwide over more than a decade and a half. The latest generation of SBML is SBML Level 3, which is structured as a self-sufficient core and optional *SBML Level 3 packages* that can be used to extend the core’s syntax and semantics [3]. The definition of each SBML Level 3 package is written in a formal specification document that is made freely available [4, 5].

SBML packages are produced and ratified according to an explicit SBML development process (http://sbml.org/Documents/SBML_Development_Process). This process also defines the content that each specification document should contain. However, it is challenging for prospective specification creators to generate a clear and complete specification document from scratch. The availability of an existing framework provides a starting point and helps authors in several respects:

- It helps ensure that required information is not forgotten from the document.
- It saves writing time by providing well-tested commands for formatting elements that are commonly used in SBML specification documents.
- It provides a cohesive look and feel for all SBML specifications.

For these reasons, SBMLPkgSpec was developed to provide a document framework for SBML package specification documents.

## Main text

SBMLPkgSpec is a LaTeX document class [6] intended to provide a common framework for writing SBML package specifications, as well as provide a uniform look and feel for the family of SBML specifications. SBMLPkgSpec builds on a number of other commonly-available LaTeX document classes, and also defines a number of new commands, so that users of SBMLPkgSpec can focus on the essential aspects of writing clear specification documents for SBML. Among the features provided by SBMLPkgSpec are the following:

- Commands for defining the SBML package version, release date, home web page, and author list, to be printed on the document’s front page.
- Commands for defining SBML *validation rules*. A convention developed for the SBML specification documents is to define validation and consistency rules that must or should be satisfied by SBML files that conform to the specification; SBML package specifications likewise define their own validation and consistency rules, and the commands in SBMLPkgSpec provide the means for easily defining and formatting them.
- Commands for formatting the names of common SBML object classes and XML primitive data types, as well as for creating new package-specific definitions. The commands for SBML object names automatically insert hyperlinks to the sections where they are defined from wherever they are referenced within a document.
- Customized commands for cross-referencing sections, tables and figures; these are designed to produce both item number and page references that are automatically hyperlinked to the appropriate locations in the finished document. They also obey some common typographical conventions (such as the use of LaTeX ties in the appropriate locations) to save authors the trouble of remembering to use them.
- Commands for formatting SBML XML examples in a stylized fashion.
- Automatic line numbering of every line in the specification document. This makes it easier to report problems and errors in specification documents, and to issue subsequent lists of errata.
- An option to print the word *DRAFT* on every page in large gray type.
- Commands for different kinds of document notes: notices (with a hand pointer in the left margin), warnings (with a red warning sign in the left margin), and reader notes (formatted as yellow rectangular notes shown in the left margin when the document is formatted in draft mode).
- Other miscellaneous features, such as a number of predefined color names.

**Fig. 1:**
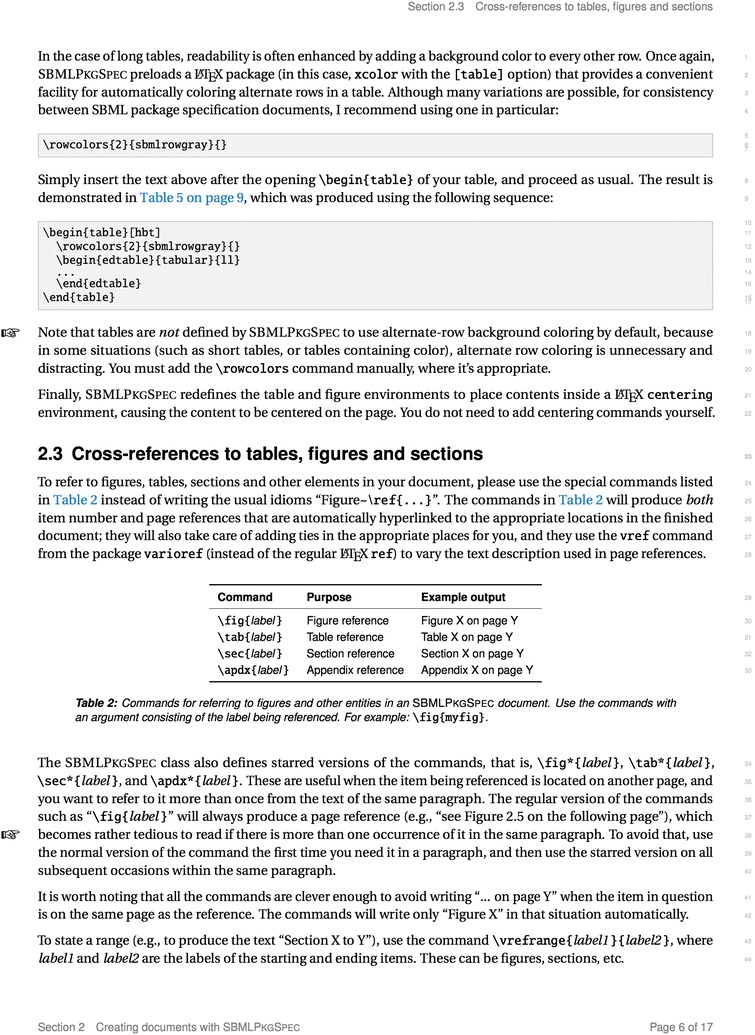
A sample page from the SBMLPkgSpec user’s guide, illustrating the look and feel of the document and some of its features

To illustrate some of the features of SBMLPkgSpec, Fig.  1 shows an image of a page from the user’s guide. It illustrates the general look of the document and some of the commands it provides.

### Installation and configuration

The use of SBMLPkgSpec should require only a recent and relatively complete installation of LaTeX2e. It was developed and tested with the TeX Live 2011 and 2016 distributions on Mac OS X 10.6–10.11 system, and has been reported to work with TeX Live on Windows and Ubuntu Linux. (For Ubuntu, make sure to install the following packages: “texlive”, “texlive-latex-extra”, “texlive-humantities”, and “texlive-fonts-extra”.) To use SBMLPkgSpec, you will need to inform your copy of LaTeX where to find the file sbmlpkgspec.cls and its accompanying subdirectory “logos”. This can be done in a variety of ways. Here are two common approaches:

- *Per-document installation* This is probably the simplest approach, although it results in multiple copies of the files. Download the SBMLPkgSpec release from the GitHub repository (https://github.com/sbmlteam/sbmlpkgspec), copy the contents (specifically, sbmlpkgspec.cls and the folder named “logos”) to the folder where you keep the other files for the SBML Level 3 package specification you are authoring, and you are done. The next time you run LaTeX in that folder, it will find the .cls file in the current directory.
- *“Central” installation* In this approach, you install sbmlpkgspec.cls in a folder where you keep other LaTeX class files, and configure your copy of LaTeX to find things there. Configuring a TeX system in this way on Unix-type systems (Linux, etc.) usually requires setting the environment variable TEXINPUTS and possibly others. Please consult the documentation for your TeX installation to determine how to do this.

Once SBMLPkgSpec is installed, users can write specification documents with the standard documentclass command in LaTeX to declare the use of the class sbmlpkgspec, and write their document using whatever editing environment they prefer, including online shared LaTeX editing environments.

### Documentation

SBMLPkgSpec comes with a complete user’s guide. Users of SBMLPkgSpec are strongly urged to read the guide; it explains everything needed to know to use the document class, and includes tips for how to make the most of it.

### Discussion

LaTeX [6] is a popular document production system in science. In systems biology and the SBML-using community, it is so popular that some software tools have been designed to produce LaTeX output directly [7, 8]. LaTeX provides tremendous power to authors, but it is also relatively difficult to use. Defining new styles is specially difficult, and requires arcane knowledge and significant patience. By simplifying the requirements for producing templated documents and providing a ready-to-use LaTeX style, SBMLPkgSpec can make it easier for SBML specification authors to use LaTeX to produce documents with a uniform format. This in turn permits authors of SBML specifications to concentrate on the technical aspects of the work.

## Limitations

SBMLPkgSpec imports many other LaTeX classes when it is used with LaTeX. These additional classes must be installed on the user’s computer for SBMLPkgSpec to work. The classes are present in many full-featured TeX distributions such as the TeX Live 2011 and 2016 distributions, but if they are not, the user will need to find and install them separately. The relevant software packages for Ubuntu Linux are noted above.

## Abbreviations

SBML: Systems Biology Markup Language
XML: Extensible Markup Language
